# An exploratory study comparing the social behavior of intersex and female goats

**DOI:** 10.3389/fvets.2026.1895618

**Published:** 2026-08-13

**Authors:** Océane Schmitt, David Schmitz-Lamping, Gesine Lühken, Gerriet Trei, Uta König von Borstel

**Affiliations:** 1 Animal Husbandry, Behaviour and Welfare, Institute of Animal Breeding and Genetics, Justus-Liebig University of Giessen, Giessen, Germany; 2 Fachgebiet Ökologische Tierhaltung, Hochschule für nachhaltige Entwicklung Eberswalde (FH), Eberswalde, Germany; 3 Animal and Pathogenetics, Institute of Animal Breeding and Genetics, Justus-Liebig University of Giessen, Giessen, Germany

**Keywords:** animal welfare, hierarchy, hornlessness, intersexuality, social network analysis

## Abstract

**Introduction:**

Goats are highly social animals that establish complex hierarchical and affiliative relationships within their herds. Understanding these social dynamics is fundamental to optimize animal welfare in sustainable livestock systems. In goats, a natural genetic variant causes dominant absence of horns (polledness). As this variant also has an influence on sex development (polled intersex syndrome, PIS), breeding for polledness can result in a higher proportion of intersexual goats in a flock. However, little is known about the behavioral roles and social integration of intersex goats within herds.

**Methods:**

This study compares the social behavior in a closed group of intersex (*n* = 7) and female (*n* = 17) polled goats to identify differences in agonistic and affiliative interactions and their implications for herd social dynamics. Behavioral data were obtained through continuous video observation and coded using the software BORIS. Interactions were analyzed via social network analysis (SNA) and hierarchical rank assessment based on David's Score.

**Results:**

Intersex goats showed a significantly higher frequency of affiliative interactions compared with females, suggesting enhanced social connectedness. No significant difference was found in agonistic interaction frequency, but intersex goats fought with significantly fewer opponents than female goats.

**Discussion:**

Although the analysis should be repeated in other herds, these findings offer novel insights into the social behavior of intersex goats and suggest that they may contribute to social cohesion rather than conflict in a herd.

## 1 Introduction

Goats are highly social animals living in herds and form complex social relationships established through affiliative and agonistic behaviors (1). Understanding these social dynamics is not only of ethological interest but also crucial for welfare-oriented management. Affiliative behaviors, such as allogrooming, rubbing or resting together, strengthen social bonds and reduce the likelihood of agonistic behaviors (1, 2). Goats show preferences for particular social partners, especially during resting when they often lie beside individuals with similar rank or temperament (3). Agonistic behavior, such as threat displays [i.e., lowering the head or displaying horns intimidate rivals without physical contact (1)] and headbutts, serves to establish and maintain dominance hierarchies within the herd, and determine the order of access to resources (1, 4–6). For example, low-ranking goats tend to feed at night when higher-ranking animals are resting, thereby avoiding conflict (4). Both agonistic and affiliative behaviors have been shown to be influenced by horn status and sex, amongst other factors (e.g., stocking density). For instance, horned goats display more non-physical threats and tend to form more stable hierarchies, while hornless goats engage more often in physical interactions, suggesting a looser or less clearly defined dominance structure (7). Intervening in conflicts, i.e., an affiliative behavior performed to disrupt conflicts, increases bonds [macaques: Petit and Thierry (8)] and herd stability [goats: Schino (3); Stanley and Dunbar (9)], and occurs more in horned herds than in hornless herds (10).

The disbudding of goats is a controversial topic in livestock production. While it may appear practical for management or to reduce injury risk (11), the procedure raises not only welfare but also ethical and legal concerns due to the pain caused to the animals, especially in goats (12). Consequently, breeding for natural, genetic hornlessness (termed “polledness”) appears to be an alternative to dehorning. The absence of horn growth in goats is caused by a complex genetic variant on caprine chromosome 1 (13, 14). However, in contrast to cattle and sheep, dominantly inherited polledness in goats is associated with recessive intersexuality (so-called “polled intersexuality syndrome, PIS”). Homozygous polled goats with XX karyotype are infertile intersexes. The phenotype of affected animals can vary regarding external appearance as well as the reproductive organs from “typically female” to “typically male”, including all possible combinations in between (13, 15). This was reflected in the measured concentrations of anti-Müllerian hormone in the gonadal tissue of homozygous hornless XX goat fetuses, which varied within the range of values seen in normal female or male animals, or were even higher than those in males (16). Additionally, blood testosterone levels in intersex goats can be comparable to those of males and accompanied by masculine behaviors such as interest in oestrus (mounting attempt, perineal nosing, foreleg striking) and mild aggression toward bucks (17). This feature of genetic hornlessness in goats is challenging its practical use as an alternative to dehorning.

While there has been some investigation comparing the behavior of hornless/horned goats [e.g., (18)], particularly with regard to dominance structures, group organization and behavior at resource sites, differences due to PIS status have hardly been investigated. This study therefore aims to contribute to filling this research gap by analyzing the social behavior (including both affiliative and agonistic behaviors) of intersex goats in comparison with female goats within a stable herd context. We expected intersex goats to have a behavioral profile closer to male goats, compared to female goats, i.e., that they would engage in more agonistic behaviors, especially amongst each other.

## 2 Materials and methods

### 2.1 Ethics approval

The study was examined and approved by the Ethics Board of the Justus Liebig University Giessen (approval number: JLU_kTV_4_2024). The herd management followed standard husbandry principles and complied with EU Regulation (EU) 2018/848 and the Animal Welfare Act of Germany.

### 2.2 Animals and housing conditions

The study was conducted on a polled herd of Saanen Goats maintained at the Oberer Hardthof research facilities of the Justus Liebig University Giessen (Germany), Institute of Animal Breeding and Genetics.

**Figure 1:**
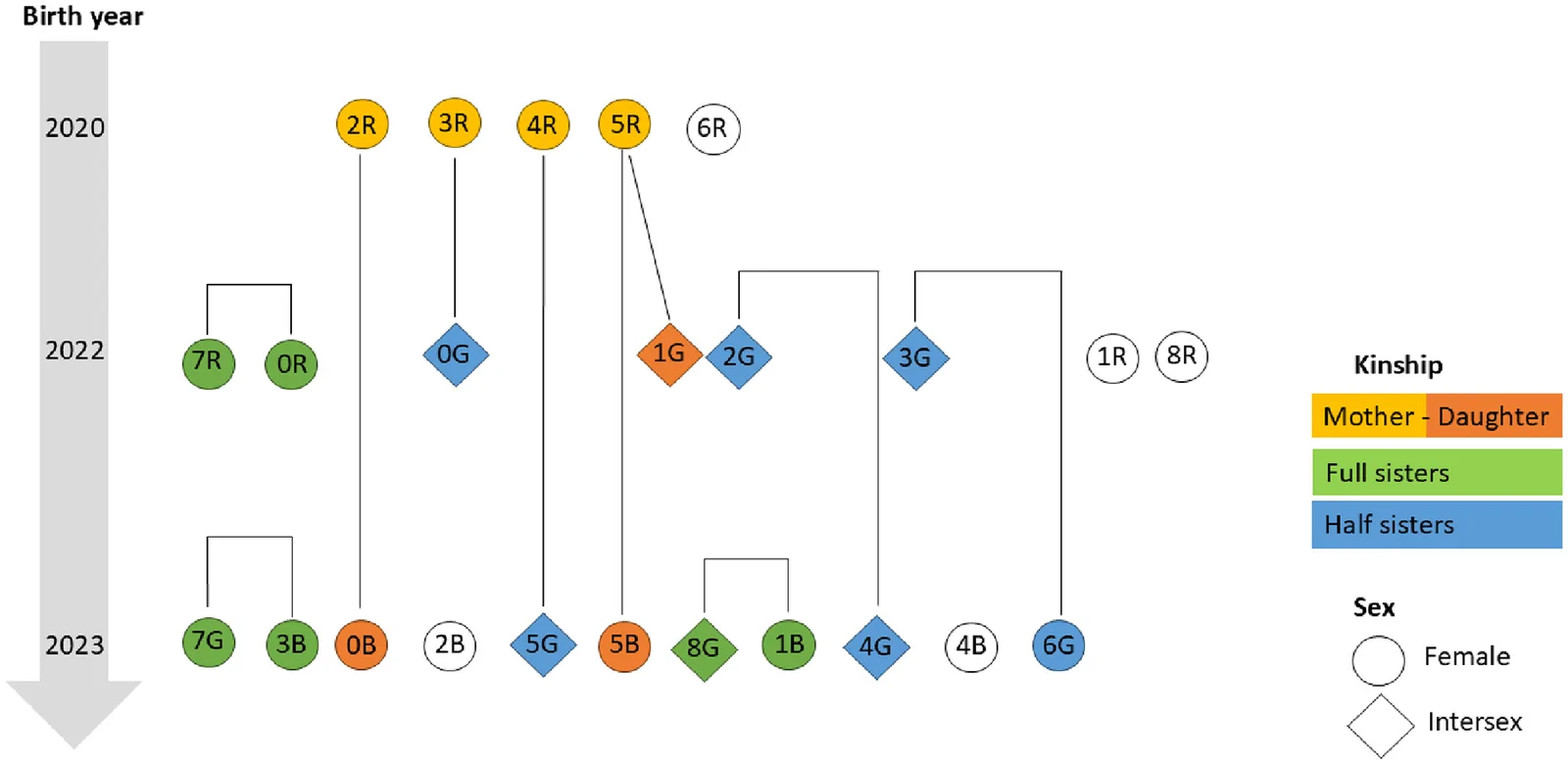
Schematic representation of the herd composition in terms of sex groups (females depicted by squares and intersex by rounds), age groups (year of birth) and kinship (mothers are in yellow, daughters in orange, full sisters in green and half-sisters in blue).

The herd consisted of 24 goats, including 17 females (XX, heterozygously polled) and 7 intersex (XX, homozygously polled) individuals. The genetic sex and horn status was determined in another project by genotyping as described elsewhere (14). The goats were aged between 1 and 4 years and some were related to each other (mother/daughter; sisters) (Figure 1). There was an age imbalance between intersex and female goats, as there was no 4 year-old intersex goat in the herd. The group was deemed stable since no alteration in its composition occurred for at least 3 months. The goats were housed in an open deep-litter barn (76 m^2^) with permanent access to pasture (450 m^2^). A wooden platform was present in the barn to facilitate climbing behavior. Hay was provided *ad libitum* via a hay dispenser, while pelleted concentrate was manually offered once daily at 08:00 h through a feeding trough. The animals had continuous access to fresh water through automated drinking throughs.

Individual identification was achieved by marking each goat on both flanks (combination of number 0–8 and color: green, red or blue).

### 2.3 Behavioral observations

**Figure 2:**
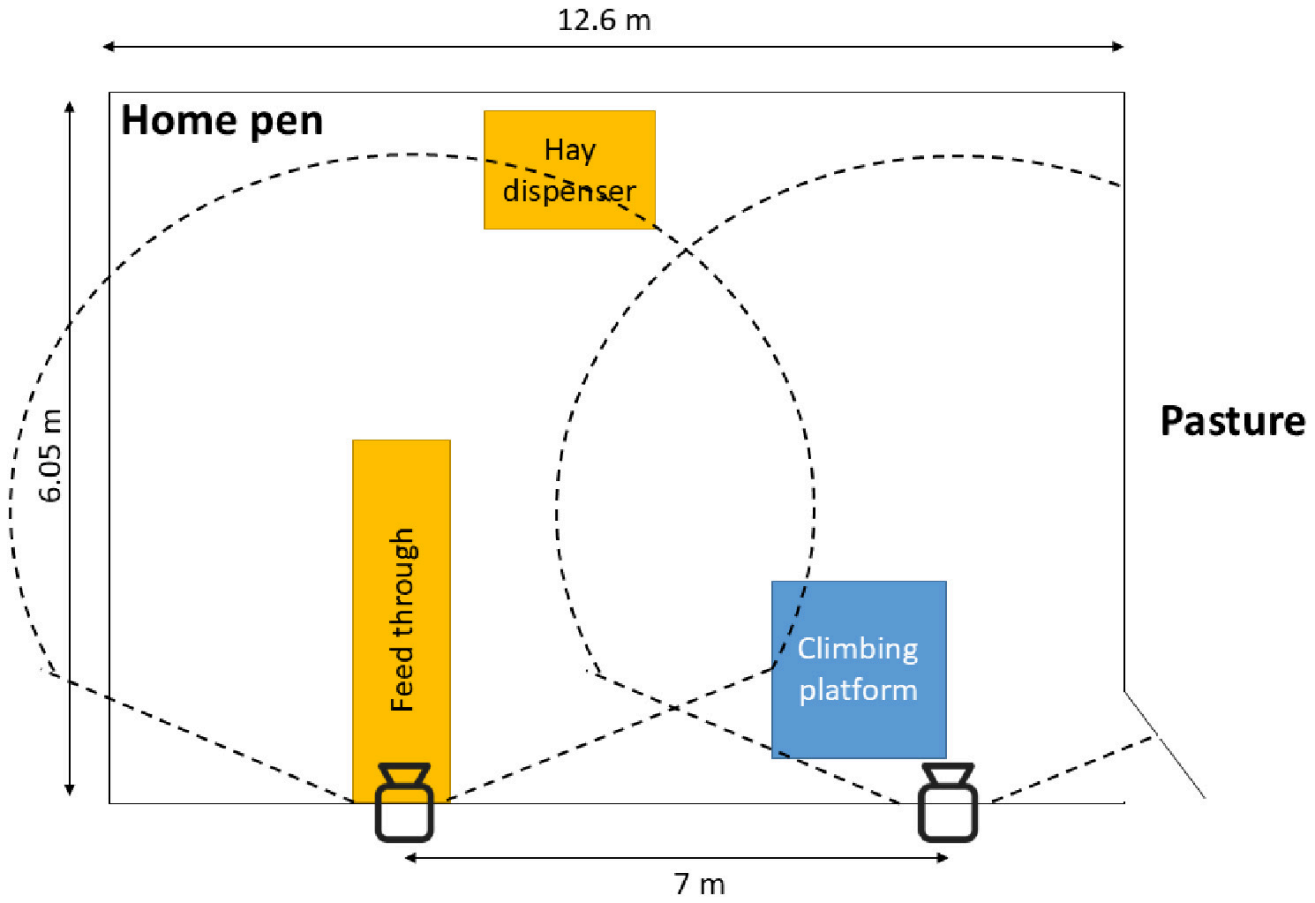
Scheme of the goat barn and details of the fields of view of the cameras.

Video recordings were obtained from two Arlo Go 2 LTE motion-sensitive cameras (Verisure Arlo Europe DAC, Ireland) positioned in the home pen to cover the feeding through (2.8 m x 0.8 m) and most of the pen, with overlapping fields of view to capture as many social interactions as possible (Figure 2). Unfortunately, part of the hay dispenser area could not be covered and thus interactions at this area (presumably mostly agonistic) could have been missed.

Recordings were made continuously throughout the day and night (24-h recording) over 3 weeks, as part of another study, and videos acquired on days when the herd was undisturbed by the presence of experimenters (17th, 18th, 28th June and 2nd−3rd July 2024) were analyzed. Camera recording was triggered and maintained by movement, producing 5-min clips as long as movement was detected. For active time behavioral observation, video clips were combined per hour, making observation material lasting 15–45 min. For feeding time behavioral observations, video clips acquired at the time of feeding (around 8:00 h) were combined into 15-min videos on average (range: 10–23 min), covering the whole feeding time (from positioning of the goats at the feeder until most goats left the feeding area). In total, 914 min (15.24 h) of active-phase recordings, covering the period between 06:00 h and 18:00 h, and 97 min (1.63 h) during feeding were analyzed.

Videos were analyzed by a single trained observer (DL) using BORIS [Behavioral Observation Research Interactive Software; (19)]. Observed behaviors (Table 1) were categorized as affiliative or agonistic, and the identity of the performer (initiating individual) and of the receiver (target individual) were recorded.

**Table 1:** Ethogram used for the observations of social behaviors in goats (2, 6).

Behavior type	Behavior	Operational definition
Agonistic	Displacement	Performer goat threatens (i.e. lowers her head and presents her “horns” to another goat) or drives another away without physical contact.
	Head-butting	Performer strikes another goat with the head.
	Pushing	Performer shoves another goat using the head or body.
	Biting	Performer's mouth makes a quick contact with another goat's body part.
Affiliative	Licking	Performer licks or grooms another goat.
	Rubbing	Performer rubs head or neck gently against another goat.
	Lying together	Two goats rest in physical contact; the performer was the goat which actively lied down next to another goat.

### 2.4 Statistical analysis

Data were analyzed using RStudio v2026.01.1 (20). Data was handled using the package tidyverse version v2.0.0 (21) and graphs were created using base R and the package RColorBrewer v 1.1.3 (22).

Linear models, from the package lme4 v 1.1.35.5 (23), were used to investigate the effect of sex (female vs. intersex) and age on interactions variables, social network metrics, and David's scores.

The threshold for significant differences was *P* < 0.05. Because this study is exploratory and was performed on a small sample size, *P*-values were not adjusted for multiple testing but bootstrapping of social network metrics allowed to assess the reliability of our conclusions (see below Section 2.4.1). Unless stated otherwise, results are presented as means ± standard error (SE).

#### 2.4.1 Social network analysis (SNA)

##### 2.4.1.1 Networks construction

Interaction matrices were generated with each goat represented as a node and each directed interaction as an edge. For each network, weighted adjacency matrices were calculated, with edge weights representing the total number of interactions from one individual to another. Separate networks were constructed for affiliative and agonistic behaviors. Network metrics were calculated using the packages igraph v 2.1.4 (24) and sna v2.8 (25).

Node-level (individual) network metrics included in the analysis:

- Degree centrality: total number of interaction partners (in + out degree).
- Betweenness centrality: frequency with which an individual lies on the shortest paths between all other pairs of individuals.
- Closeness centrality: the inverse of the average shortest path length from an individual to all others.

##### 2.4.1.2 Bootstrapping of network metrics

Bootstrap confidence intervals (*n* = 1,000 iterations) for all dyadic and individual metrics were generated to quantify uncertainty around network measures. This was performed by resampling the interaction dataset with replacement, reconstructing the network for each bootstrap iteration, and recalculating the dyadic edge weights, the individual centrality indices and the global clustering coefficient.

Confidence intervals around SNA metrics were generally wide, reflecting variability. A few dyads (affiliative = 4/57 dyads, agonistic = 5/104 dyads) exhibited lower confidence limits above zero and a confidence interval (relative to the mean) below 2 for the frequency of interactions, indicating stable relationships.

##### 2.4.1.3 Testing for non-random social structure

A permutation-based null model was used to evaluate whether observed interaction patterns differed from random expectation. The network was permutated by randomly shuffling “receiver” identities while keeping “performer” identities fixed, which preserved the number of interactions performed by each individual and the overall frequency of interaction events, but removed partner choice.

The network metric of interest (e.g., mean dyadic edge weight, degree centrality) was recalculated for each permutation, generating a null distribution of 1,000 permuted values. The observed metric was compared to this null distribution, and significance was assessed as per Equation 1:


$$\begin{array}{l} p=\frac{number\;of\;permuted\;values\;\geq \;observed\;value}{1,000} \end{array}$$ (1)

Given that for both the affiliative and negative networks, the observed value significantly exceeded the 95 % interval of the null distribution, we concluded that interactions inside each network were more structured than expected by chance, indicating non-random partner choice.

#### 2.4.2 Dominance hierarchy

The David's Score (26) was applied to quantify dominance order during feeding phase. To meet gaussian distribution requirement, an inverse hyperbolic sine transformation was done on the David's Score before analyzing the effects of sex and age with linear models. Estimated means and standard errors reported are back-transformed.

## 3 Results

### 3.1 Social networks

#### 3.1.1 Overview

A total of 259 interactions were recorded from the “active time” videos, including 162 agonistic and 97 affiliative interactions (Supplementary Tables S1, S2). Among agonistic behaviors, head-butting (44.4 %) and pushing (40.7 %) were most frequent, while biting (14.2 %) and displacements (0.6 %) occurred less often. Lying together (54.6 %) and rubbing (40.2 %) were the most commonly performed affiliative behaviors, while licking (3.1 %) and conflict intervention (2.1 %) were quite rare.

From the feeding time, 150 interactions were observed, all being agonistic interactions.

Network analyses and centrality stability assessment showed distinct structural characteristics between affiliative and agonistic interactions (Table 2). The median degree centrality was higher in the agonistic than in the affiliative network (5 vs. 2; *W* = 423.5; *P* = 0.001) but the median closeness centrality was higher in the affiliative network than in the agonistic network (0.025 vs. 0.009, *W* = 0; *P* < 0.0001). There was no difference between the network in the median betweenness centrality (agonistic = 89.3, affiliative = 81.8, *W* = 320; *P* = 0.35).

**Table 2:** Characteristics of social networks made from affiliative behaviors (“affiliative network”) or agonistic behaviors (“agonistic network”).

Network metric	Affiliative network	Agonistic network	Chi-square value df = 1	*P*-value
Degree centrality	8.4 ± 1.35	13.5 ± 1.43	6.38	0.01
Betweenness	36.8 ± 9.73	26.1 ± 5.90	0.02	0.90
Closeness	0.06 ± 0.045	0.02 ± 0.000	8.53	0.003

Sex and age effects on the social network metrics are presented below, details of the model outputs (Supplementary Table S3) and individual values in the social networks (Supplementary Table S4) can be found in Supplementary material.

#### 3.1.2 Sex differences

**Figure 3:**
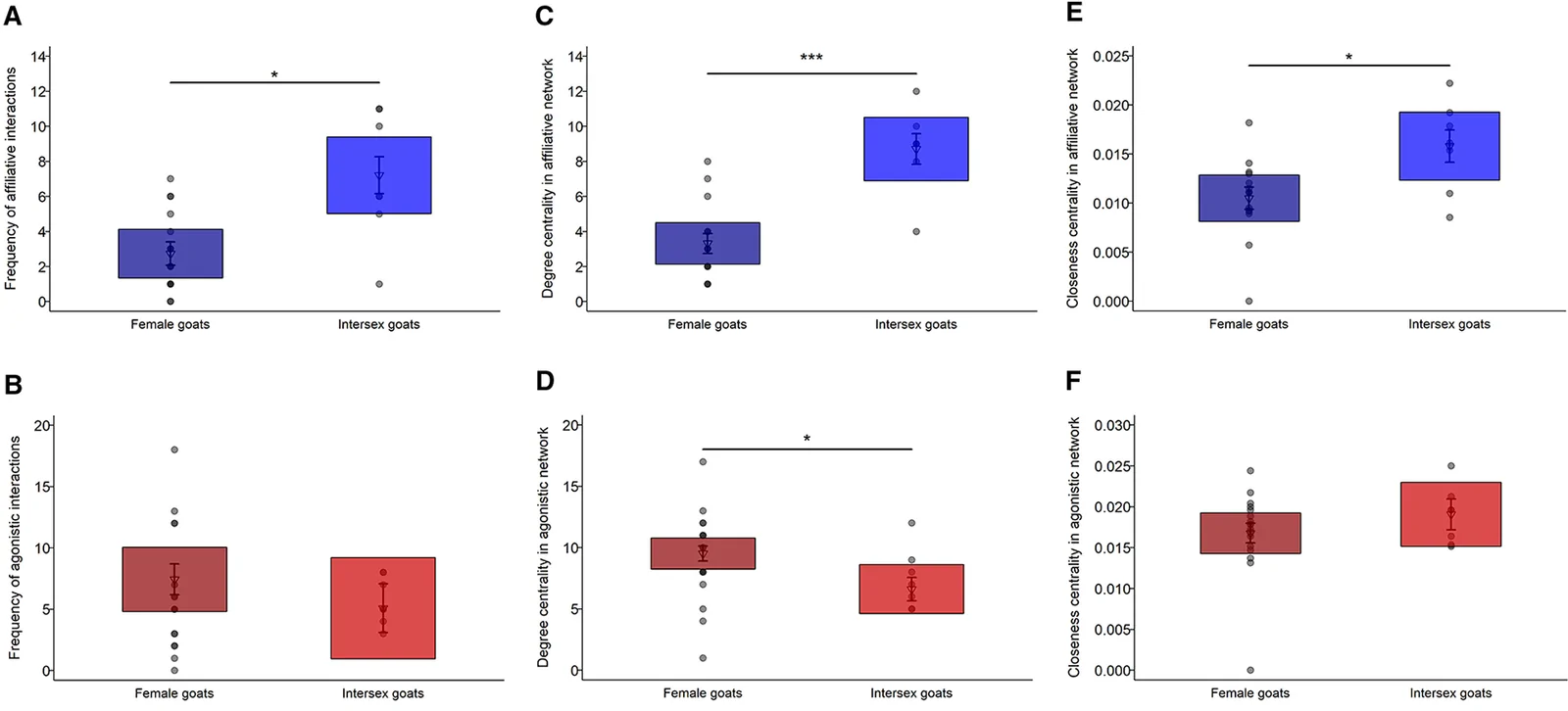
Differences between female and intersex goats in terms of: Frequency of affiliative behaviors **(A)** and agonistic behaviors **(B)** observed; Degree centrality in the affiliative **(C)** and agonistic **(D)** networks; Closeness centrality in affiliative **(E)** and agonistic **(F)** networks. Stars above boxes represent a statistically significant difference between the female and intersex goats at the thresholds: *P < 0.05; ***P < 0.0001.

**Figure 4:**
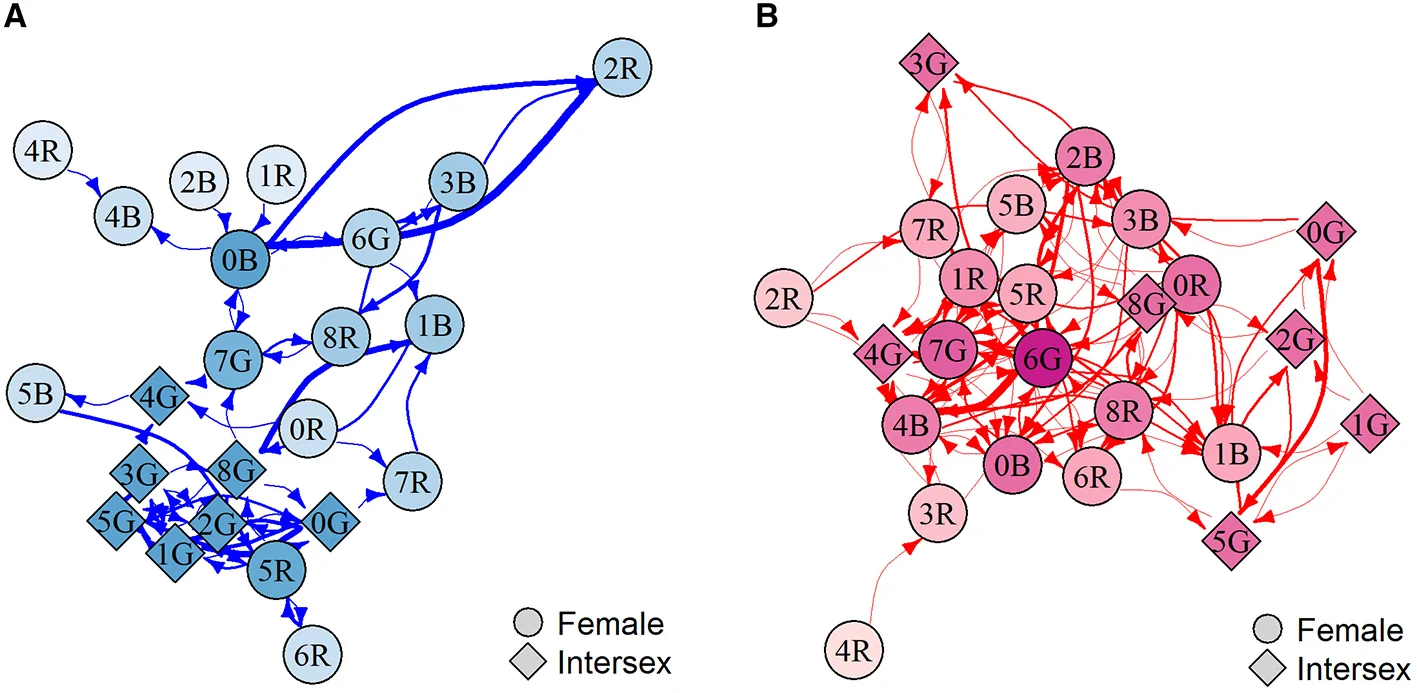
Graphical representations of the social networks built from the analysis of affiliative **(A)** and agonistic **(B)** interactions in a stable herd of 24 hornless goats. Intersex goats (*n* = 7) are represented by circles, and female goats (*n* = 17) are represented by squares. The association of number and letter indicate the goat ID. Interactions are represented by directional arrows (from the performer to the receiver), thicker arrows indicate greater number of interactions between the two individuals.

Intersex goats displayed affiliative behaviors significantly more frequently than females (7.2 ± 1.18 vs. 2.8 ± 0.70, respectively; *t* = 3.2, *P* = 0.0045) (Figure 3A). No significant difference was found in the frequency of agonistic interactions between intersex and female goats (3.2 ± 2.00 vs. 7.2 ± 1.18, respectively; *t* = −1.75, *P* = 0.095) (Figure 3B). There was a strong preference for individuals of the same sex to interact with each other, whether it was for affiliative ($X_{1}^{2}$ = 38.4, *P* < 0.0001) or agonistic behaviors ($X_{1}^{2}$ = 16.6, *P* < 0.0001) (Supplementary Table S5 and Figure 4).

Compared to female goats, the degree centrality of intersex goats was higher in the affiliative network (8.7 ± 1.00 vs. 3.3 ± 0.61; *t* = 4.74, *P* = 0.0001; Figure 3C) and lower in the agonistic network (5.8 ± 1.08 vs. 8.7 ±0.64; *t* = −2.32, *P* = 0.03; Figure 3D).

Closeness centrality was higher for intersex goats than for female goats in the affiliative network (0.016 ± 0.002 vs. 0.011 ± 0.001; *t* = 2.44, *P* = 0.025; Figure 3E), but there was no sex difference in the agonistic network (females: 0.017 ± 0.001, intersex: 0.018 ± 0.002; *t* = 0.51, *P* = 0.61; Figure 3F).

The betweenness centrality was not influenced by sex for the affiliative network (*t* = 1.14, *P* = 0.27) or the agonistic network (*t* = 0.18, *P* = 0.86).

#### 3.1.3 Age differences

Age did not influence the metrics of the affiliative network or the frequency of affiliative interactions, but it influenced some of the metrics of the agonistic network (Supplementary Table S3).

The frequency of agonistic interactions performed was higher for younger goats, compared to the older goats (age 1: 6.1 ± 1.52 vs. age 4: 0.6 ± 2.42, *t* = 2.9, *P* = 0.025).

The degree centrality in the agonistic network was lowest for older goats (age 4: 3.7 ± 1.31), compared to goats aged 1 year-old (10.1 ± 0.82, *t* = 4.4, *P* = 0.0007), and 2 year-old (8.0 ± 0.90, *t* = 2.7, *P* =0.037).

### 3.2 Hierarchy

The hierarchy analysis revealed the David's score was significantly lower for 1 year-old goats (−2.6 ± 0.77) compared to 2 year-old goats (2.4 ± 0.85; *t* = 4.3, *P* = 0.0003) and to 4 year-old goats (2.0 ± 1.22; *t* = 3.5, *P* = 0.002). However, David's score did not differ significantly between females and intersex goats (0.8 ± 0.60 vs. 0.39 ± 1.01, respectively; *t* = 0.3, *P* = 0.74), mostly due to large intra-group variations.

## 4 Discussion

This study provides the first comparison of the social behavior of intersex and female goats. Contrary to our initial hypothesis, intersex goats performed affiliative behaviors significantly more frequently than female goats, and were more connected to other goats in the affiliative network. Although they did not differ in the frequency, intersex goats performed agonistic behaviors with fewer different partners than female goats. Therefore, intersex goats seem to adhere to the ‘affiliative identity profile' described in herds of female dairy goats (27). The individuals in this profile were characterized by a low level of agonistic interactions initiated and received, high level of affiliative interactions initiated, highest level of cortisol, and the longest solving time in the first exposure to a T-maze and an intermediate time for the second exposure (i.e., greatest performance improvement) (28). However, it also became apparent from the social networks that social interactions, whether affiliative or agonistic, where preferably performed between individuals of the same “phenotypical” sex (i.e., females together and intersex together), for reasons that are unknown and could not be identified in this study.

Overall, more agonistic than affiliative behaviors were observed during the observation period, making the agonistic network denser. In the agonistic network, individuals interacted with many other individuals (high degree centrality) but agonistic interactions were not structured into a cohesive network (low closeness), which suggests opportunistic aggression rather than bullying amongst group members (29). The affiliative network was characterized by fewer partners (low degree centrality) and greater cohesiveness (high closeness centrality), suggesting preferential affiliative bonding (29).

Intersex goats had a higher degree of centrality (i.e., more interaction partners) in the affiliative network and lower in the agonistic network. This suggests that intersex goats are widely social and engage in affiliative behavior with several members of their social group, but are selective for the agonistic behaviors, which they perform with fewer individuals. In other words, this means that intersex goats in our study had “many friends and few enemies”. The elevated affiliative activity among intersex goats may reflect enhanced social motivation or a compensatory behavioral strategy promoting group acceptance, for instance by adopting an “affiliative” identity profile (27, 28).

Despite expectations that intersex goats might display more agonistic behaviors, given that they often exhibit elevated testosterone levels similar to males (17), no significant difference was observed between females and intersex goats. While testosterone is commonly associated with aggression, it can also increase social engagement depending on context and dominance status (30). For example, testosterone promotes affiliative behavior in social contexts, particularly with stable social partners, in Mongolian gerbils (31). On the other hand, testosterone seems less relevant to explain the variability in cattle docility in a chute test than cortisol (32), further supporting that the influence of testosterone on behavior might be context-dependent. However, testosterone was not measured in the present study, so we encourage further studies to include such measure in order to understand whether hormonal effects manifest differently in mixed and stable social groups of goats. Similarly, the reproductive status and the associated hormonal levels (e.g., estrogen, progesterone) were not assessed in the current study, although they might affect the social behavior of goats (but this remains unstudied).

As described in other papers (14, 17), some of the intersex goats in the current study were bigger than the female goats and had masculine attributes (e.g. bigger beard; Supplementary Figures S1, S2), however, they were not higher in the hierarchy ranking. Individuals occupying intermediate positions in the social structure may facilitate cohesion by interacting across hierarchical levels, consistent with findings from social network studies showing that certain individuals act as connectors or “brokers” linking subgroups and maintaining group cohesion (33, 34). This hypothesis seems supported by the fact that intersex goats occupied central bridging positions within affiliative networks (as shown by betweenness centrality values higher than the median), connecting subgroups that were otherwise weakly linked. In the current study, intersex goats did not differ from female goats in their betweenness centrality, but the most connected goat was an intersex goat (8G) and the betweenness centrality value was above the median for 5 out of 7 intersex goats. Together, our results suggest that intersex goats may play a stabilizing role in maintaining herd cohesion. Only one instance of third-party intervention to interrupt a fight between two goats was observed in our study, which confirms that goats also perform this behavior, like in other ungulate species [e.g., sheep: (35), cattle: (36)], and encourages its inclusion in social behaviors ethograms.

Finally, age differences were detected for the frequency of agonistic behaviors, with younger goats performing more agonistic behaviors and with more partners than older goats, and hierarchy rank, with younger goats having lower ranks in the hierarchy than older goats. Another study observed that older goats ranked higher in the hierarchy, in agreement with our results, but they also fought more than younger goats, in contrast to our results (6). However, the effect of age on the performance of agonistic interactions was found only in horned goats and not in dehorned goats (6), suggesting an interplay between horn status and age on agonistic behavior and hierarchy ranking. Studies on mountain goats are also contradictory as some found no effect of age on hierarchy rank (37) and others found a positive correlation between age and hierarchy rank (the higher the more dominant) (38). Such contradicting results both in wild and domestic populations of goats suggest that the multiple factors determining hierarchy ranks might have different influence in different herds. Comparisons with previous studies should be interpreted with caution because the age structure differed substantially between herds. For instance, Górecki et al. (6) studied goats ranging from 1 to 11 years of age, whereas in the present study the age of goats was not balanced across sexes and there was no intersex goat aged 4 years old. Furthermore, the kinship structure of the present herd must also be considered when since mother-offspring relationships might be different than relationships between unrelated individuals. For instance, kinship status affected affiliative interactions, as mother-offspring pairs groomed each other more, but not agonistic interactions of ring-tailed coatis (39). While such detailed information are not available in goats, adult female mountain goats were observed to associate (i.e., stay with 100 m of each other) preferably with their mother, or with females with the same reproductive status (40).

While the current study provides novel insights, several limitations should be acknowledged. The sample size was small, with an uneven number of intersex and female goats and uneven age distribution, and restricted to a single herd under specific management conditions, which may limit generalisability. Furthermore, there were some methodological limitations, such as the blind spot in the camera set-up which might have caused interactions at the hay dispenser to be missed in the video analysis. Therefore, future research should examine additional and larger populations, include physiological measures such as hormone profiles, and include more affiliative behaviors such as proximity. In addition, the observed herd was not used for milk production, thus it would be interesting to explore the social dynamics of intersex goats in commercial herds, in which husbandry practices and different herd sizes might result in different social dynamics. Furthermore, longitudinal studies, including possible seasonal effects, would help determine whether intersex goats maintain their intermediary social roles over time. Finally, this study only looked at genetically female goats, thus the social behavior of intersex goats in genetically male only herds and in mixed herds would be beneficial to get a complete understanding of the factors affecting social dynamics of intersex goats.

## 5 Conclusion

The results of this study must be considered very cautiously, given that they are based on a single herd with a specific social and environmental conditions that might not be representative of commercial herds. Intersex goats in this study performed affiliative behaviors more frequently and with more partners, indicating higher sociality. Furthermore, contrary to our initial hypothesis, intersex goats performed agonistic behaviors as frequently but with fewer partners than female goats. Hierarchy ranks were not affected by whether the goat was female or intersex. From a welfare perspective, the presence of intersex goats in hornless herds does not seem detrimental to social stability. On the contrary, their higher engagement in affiliative behaviors and intermediate hierarchy status may contribute to reducing conflict and improved cohesion in the herd, provided that environmental enrichment and sufficient space are provided.

## Data Availability

The datasets presented in this study can be found in online repositories. The names of the repository/repositories and accession number(s) can be found below: Open Science Framework repository (https://osf.io/q4b9g?view_only=b1d05b2b255a4c07893458f05d7cef7d).

## References

[1] Miranda-de La Lama GC Mattiello S. The importance of social behaviour for goat welfare in livestock farming. Small Rumin Res. (2010) 90:1–10. doi: 10.1016/j.smallrumres.2010.01.006

[2] Andersen IL Tønnesen H Estevez I Cronin GM Bøe KE. The relevance of group size on goats' social dynamics in a production environment. Appl Anim Behav Sci. (2011) 134:136–43. doi: 10.1016/j.applanim.2011.08.003

[3] Schino G. Reconciliation in domestic goats. Behaviour. (1998) 135:343–56. doi: 10.1163/156853998793066302

[4] Andersen IL Bøe KE. Resting pattern and social interactions in goats—the impact of size and organisation of lying space. Appl Anim Behav Sci. (2007) 108:89–103. doi: 10.1016/j.applanim.2006.10.015

[5] Barroso FG Alados CL Boza J. Social hierarchy in the domestic goat: effect on food habits and production. Appl Anim Behav Sci. (2000) 69:35–53. doi: 10.1016/S0168-1591(00)00113-1 10856783

[6] Górecki MT Sochacka J Kazmierczak S. Dominance hierarchy, milking order, and neighbour preference in domestic goats. Small Rumin Res. (2020) 191:106166. doi: 10.1016/j.smallrumres.2020.106166

[7] Tölü C Savaş T. A brief report on intra-species aggressive biting in a goat herd. Appl Anim Behav Sci. (2007) 102:124–9. doi: 10.1016/j.applanim.2006.03.002

[8] Petit O Thierry B. Aggressive and peaceful interventions in conflicts in Tonkean macaques. Anim Behav. (1994) 48:1427–36. doi: 10.1006/anbe.1994.1378

[9] Stanley CR Dunbar RIM. Consistent social structure and optimal clique size revealed by social network analysis of feral goats, Capra hircus. Anim Behav. (2013) 85:771–9. doi: 10.1016/j.anbehav.2013.01.020

[10] Keil NM Sambraus HH. “Intervenors” in agonistic interactions amongst domesticated goats. Z Säugetierkd. (1998) 63:266–72.

[11] Waiblinger S Schmied-Wagner C Mersmann D Nordmann E. Social behaviour and injuries in horned and hornless dairy goats. In: *Proceedings of the XVth International Congress of the International Society for Animal Hygiene*. Presented at the Animal hygiene and sustainable livestock production, Vienna, Austria. Brno: Tribun EU (2011).

[12] Hempstead M Waas J Stewart M Sutherland M. Goat kids are not small calves: species comparisons in relation to disbudding. Anim welf. (2020) 29:293–312. doi: 10.7120/09627286.29.3.293

[13] Pailhoux E Vigier B Chaffaux S Servel N Taourit S Furet J-P . A 11.7-kb deletion triggers intersexuality and polledness in goats. Nat Genet. (2001) 29:453–8. doi: 10.1038/ng769 11726932

[14] Simon R Lischer HEL Pieńkowska-Schelling A Keller I Häfliger IM Letko A . New genomic features of the polled intersex syndrome variant in goats unraveled by long-read whole-genome sequencing. Anim Genet. (2020) 51:439–48. doi: 10.1111/age.12918 32060960

[15] Pannetier M Elzaiat M Thépot D Pailhoux E. Telling the story of XX sex reversal in the goat: highlighting the sex-crossroad in domestic mammals. Sex Dev. (2012) 6:33–45. doi: 10.1159/000334056 22094227

[16] Pailhoux E Vigier B Vaiman D Servel N Chaffaux S Cribiu EP . Ontogenesis of female-to-male sex-reversal in XX polled goats. Dev Dyn. (2002) 224:39–50. doi: 10.1002/dvdy.10083 11984872

[17] Bosu WT Basrur PK. Morphological and hormonal features of an ovine and a caprine intersex. Can J Comp Med. (1984) 48:402–9. 6509368 PMC1236093

[18] Aschwanden J Gygax L Wechsler B Keil NM. Social distances of goats at the feeding rack: Influence of the quality of social bonds, rank differences, grouping age and presence of horns. Appl Anim Behav Sci. (2008) 114:116–31. doi: 10.1016/j.applanim.2008.02.002

[19] Friard O Gamba M. BORIS: a free, versatile open-source event-logging software for video/audio coding and live observations. Methods Ecol Evol. (2016) 7:1325–30. doi: 10.1111/2041-210X.12584

[20] Core Team R. R: A Language and Environment For Statistical Computing (2022).

[21] Wickham H Averick M Bryan J Chang W McGowan L François R . Welcome to the Tidyverse. JOSS. (2019) 4:1686. doi: 10.21105/joss.01686

[22] Neuwirth E. RColorBrewer: ColorBrewer Palettes (2022).

[23] Bates D Maechler M Bolker B. Fitting linear mixed-effects models using lme4. J Stat Softw. (2015) 67:1–48. doi: 10.18637/jss.v067.i01

[24] Nepusz GCAT Csárdi G. The igraph software package for complex network research. Complex Syst. (2006) 1695:1–9.

[25] Butts CT. sna: Tools for Social Network Analysis. R package (2024).

[26] Gammell MP De Vries H Jennings DJ Carlin CM Hayden TJ. David's score: a more appropriate dominance ranking method than Clutton-Brock et al.'s index. Anim Behav. (2003) 66:601–5. doi: 10.1006/anbe.2003.2226

[27] Miranda-de La Lama GC Sepúlveda WS Montaldo HH María GA Galindo F. Social strategies associated with identity profiles in dairy goats. Appl Anim Behav Sci. (2011) 134:48–55. doi: 10.1016/j.applanim.2011.06.004

[28] Pascual-Alonso M María GA Sepúlveda WS Villarroel M Aguayo-Ulloa L Galindo F . Identity profiles based on social strategies, morphology, physiology, and cognitive abilities in goats. J Vet Behav. (2013) 8:458–65. doi: 10.1016/j.jveb.2013.05.003

[29] Farine DR Whitehead H. Constructing, conducting and interpreting animal social network analysis. J Anim Ecol. (2015) 84:1144–63. doi: 10.1111/1365-2656.12418 26172345 PMC4973823

[30] Eisenegger C Haushofer J Fehr E. The role of testosterone in social interaction. Trends Cogn Sci. (2011) 15:263–71. doi: 10.1016/j.tics.2011.04.008 21616702

[31] Kelly AM Thompson RR. Testosterone facilitates nonreproductive, context-appropriate pro- and anti-social behavior in female and male Mongolian gerbils. Horm Behav. (2023) 156:105436. doi: 10.1016/j.yhbeh.2023.105436 37776832

[32] Geburt K Piechotta M König Von Borstel U Gauly M. Influence of testosterone on the docility of German Simmental and Charolais heifers during behavior tests. Physiol Behav. (2015) 141:164–71. doi: 10.1016/j.physbeh.2015.01.030 25624109

[33] Lusseau D Newman MEJ. Identifying the role that animals play in their social networks. Proc R Soc Lond B. (2004) 271:S477–81. doi: 10.1098/rsbl.2004.0225 15801609 PMC1810112

[34] Shizuka D McDonald DB. A social network perspective on measurements of dominance hierarchies. Anim Behav. (2012) 83:925–34. doi: 10.1016/j.anbehav.2012.01.011

[35] Papadaki K Laliotis GP Koutsouli P Pafilis P Bizelis I. Do sheep (*Ovis aries*) perform third-party interventions? Ethology. (2022) 128:657–67. doi: 10.1111/eth.13326

[36] Hodgson GMW Flay KJ Perroux TA McElligott AG. Strategic third-party interventions in feral ungulate affiliation. Proc R Soc B. (2025) 292:20251086. doi: 10.1098/rspb.2025.1086 40628476 PMC12308338

[37] Fournier F Festa-Bianchet M. Social dominance in adult female mountain goats. Anim Behav. (1995) 49:1449–59. doi: 10.1016/0003-3472(95)90066-7

[38] Côté SD. Dominance hierarchies in female mountain goats: stability, aggressiveness and determinants of rank. Behaviour. (2000) 137:1541–66. doi: 10.1163/156853900502718

[39] Hirsch BT Stanton MA Maldonado JE. Kinship shapes affiliative social networks but not aggression in ring-tailed coatis. PLoS ONE. (2012) 7:e37301. doi: 10.1371/journal.pone.0037301 22624010 PMC3356279

[40] Godde S Côté SD Réale D. Female mountain goats, oreamnos americanus, associate according to kinship and reproductive status. Anim Behav. (2015) 108:101–7. doi: 10.1016/j.anbehav.2015.07.005

