# Supplementary material for: An exploratory study comparing the social behavior of intersex and female goats
Source: Front Vet Sci. 2026 Aug 13;13:1895618. doi: 10.3389/fvets.2026.1895618 (PMC13520204; doi:10.3389/fvets.2026.1895618)
Supplement: Supplementary file 1 [file Table_1.docx]

Supplementary Material

**Table S1.** Number of recorded behaviours in a stable herd of 24 goats (17 females and 7 intersex). Data was obtained from 914 minutes (15.24 h) of video recordings (“active time”, between 06:00 h and 18:00 h).

| **Category** | **Behaviour** | **Number of occurrences** |
| --- | --- | --- |
| Agonistic | Biting | 23 |
| Agonistic | Displace | 1 |
| Agonistic | Head-butting | 72 |
| Agonistic | Pushing | 66 |
| Affiliative | Interrupt | 2 |
| Affiliative | Laying together | 53 |
| Affiliative | Licking | 3 |
| Affiliative | Rubbing | 39 |

**Table S2.** Number of occurrences of the recorded behaviours per goat

| **Performer** | **Biting** | **Displace** | **Head-butting** | **Interrupt** | **Laying together** | **Licking** | **Pushing** | **Rubbing** |
| --- | --- | --- | --- | --- | --- | --- | --- | --- |
| 0B | 1 | 0 | 4 | 2 | 4 | 0 | 1 | 1 |
| 0G | 0 | 0 | 6 | 0 | 4 | 0 | 2 | 6 |
| 0R | 5 | 0 | 6 | 0 | 1 | 1 | 11 | 1 |
| 1B | 0 | 0 | 0 | 0 | 2 | 0 | 0 | 0 |
| 1G | 0 | 0 | 1 | 0 | 6 | 0 | 3 | 5 |
| 1R | 5 | 0 | 3 | 0 | 1 | 0 | 5 | 0 |
| 2B | 1 | 0 | 8 | 0 | 0 | 0 | 3 | 1 |
| 2G | 0 | 0 | 3 | 0 | 5 | 0 | 2 | 1 |
| 2R | 3 | 0 | 2 | 0 | 4 | 0 | 0 | 2 |
| 3B | 1 | 0 | 2 | 0 | 4 | 0 | 4 | 2 |
| 3G | 1 | 0 | 1 | 0 | 3 | 0 | 1 | 2 |
| 3R | 0 | 0 | 0 | 0 | 0 | 0 | 3 | 0 |
| 4B | 0 | 0 | 2 | 0 | 0 | 0 | 3 | 0 |
| 4G | 0 | 0 | 4 | 0 | 0 | 1 | 4 | 0 |
| 4R | 1 | 0 | 0 | 0 | 1 | 0 | 0 | 0 |
| 5B | 1 | 0 | 1 | 0 | 1 | 1 | 1 | 1 |
| 5G | 0 | 0 | 6 | 0 | 3 | 0 | 1 | 3 |
| 5R | 1 | 0 | 0 | 0 | 0 | 0 | 1 | 5 |
| 6G | 2 | 0 | 7 | 0 | 1 | 0 | 9 | 0 |
| 6R | 1 | 0 | 1 | 0 | 2 | 0 | 0 | 1 |
| 7G | 0 | 0 | 3 | 0 | 1 | 0 | 3 | 3 |
| 7R | 0 | 1 | 1 | 0 | 1 | 0 | 3 | 1 |
| 8G | 0 | 0 | 2 | 0 | 6 | 0 | 3 | 4 |
| 8R | 0 | 0 | 9 | 0 | 3 | 0 | 3 | 0 |

**Table S3.** Estimated means and standard errors of the frequency of affiliative and agonistic behaviours and social network metrics. Statistical significance of the sex and age differences are represented by superscript letters (a, b: P < 0.05; A, B: P < 0.01).

|  | **Females** | **Intersex goats** | **1 year-old** | **2 year-old** | **4 year-old** |
| --- | --- | --- | --- | --- | --- |
| ***Affiliative Network*** | | | | | |
| Frequency | **2.8 ± 0.7 ^A^**  **[1.4 - 4.3]** | **7.2 ± 1.18 ^B^**  **[4.7 - 9.7]** | 4.7 ± 0.9  [2.9 - 6.6] | 5.1 ± 0.99  [3.1 - 7.2] | 5.2 ± 1.43  [2.2 - 8.2] |
| Degree centrality | **3.3 ± 0.61 ^A^**  **[2 - 4.6]** | **8.7 ± 1.00 ^B^**  **[6.6 - 10.8]** | 6.0 ± 0.76  [4.5 - 7.6] | 6.0 ± 0.83  [4.3 - 7.7] | 5.3 ± 1.2  [2.8 - 7.8] |
| Betweeness centrality | 11.5 ± 6.58  [1.9 - 29.4] | 31.4 ± 18.4  [4.8 - 81.5] | 47.5 ± 17.2  [18.4 - 90.2] | 14.7 ± 10.5  [0.9 - 44.9] | 7.7 ± 11  [0 - 47.8] |
| Closeness centrality | **0.01 ± 0.001 ^a^**  **[0.008 - 0.013]** | **0.02 ± 0.002 ^b^**  **[0.012 - 0.02]** | 0.013 ± 0.0016  [0.01 - 0.016] | 0.013 ± 0.0017  [0.01 - 0.017] | 0.011 ± 0.0027  [0.005 - 0.017] |
| ***Agonistic Network*** | | | | | |
| Frequency | 7.3 ± 1.18  [4.8 - 9.7] | 3.2 ± 2  [-1 - 7.4] | 6.1 ± 1.52  [2.9 - 9.3] | **9.0 ± 1.68 ^a^**  **[5.5 - 12.5]** | **0.6 ± 2.42 ^b^**  **[-4.5 - 5.6]** |
| Degree centrality | **8.7 ± 0.64 ^a^**  **[7.4 - 10.1]** | **5.8 ± 1.08 ^b^**  **[3.6 - 8.1]** | **10.1 ± 0.82 ^A^**  **[8.4 - 11.8]** | **8.0 ± 0.91 ^a^**  **[6.1 - 9.9]** | **3.7 ± 1.31 ^Bb^**  **[1 - 6.5]** |
| Betweeness centrality | 31.3 ± 8.05  [14.5 - 48.1] | 34.2 ± 13.6  [5.8 - 62.7] | 39.1 ± 10.4  [17.4 - 60.8] | 24.5 ± 11.4  [0.7 - 48.4] | 34.7 ± 16.5  [0.3 - 69] |
| Closeness centrality | 0.017 ± 0.0012  [0.015 - 0.02] | 0.018 ± 0.002  [0.014 - 0.023] | 0.016 ± 0.0015  [0.013 - 0.02] | 0.02 ± 0.0017  [0.016 - 0.023] | 0.017 ± 0.0025  [0.012 - 0.022] |

**Table S4.** Characteristics (sex and age) of the goats, number of interactions performed and social network metrics in the affiliative and agonistic networks.

|  |  |  | **Affiliative network** | | | | **Agonistic network** | | | |
| --- | --- | --- | --- | --- | --- | --- | --- | --- | --- | --- |
| **Performer** | **Sex** | **Age** | **N** | **Degree centrality** | **Betweenness centrality** | **Closeness centrality** | **N** | **Degree centrality** | **Betweenness centrality** | **Closeness centrality** |
| 0B | F | 1 | 7 | 8 | 111.3 | 0.01 | 6 | 12 | 16.2 | 0.01 |
| 0R | F | 2 | 2 | 2 | 0.0 | 0.01 | 22 | 12 | 0.0 | 0.02 |
| 1B | F | 1 | 2 | 4 | 99.8 | 0.01 | 0 | 8 | 0.0 | 0.00 |
| 1R | F | 2 | 1 | 1 | 0.0 | 0.01 | 13 | 10 | 0.2 | 0.02 |
| 2B | F | 1 | 1 | 1 | 0.0 | 0.01 | 12 | 11 | 18.2 | 0.01 |
| 2R | F | 4 | 6 | 3 | 0.0 | 0.01 | 5 | 4 | 0.0 | 0.02 |
| 3B | F | 1 | 6 | 4 | 5.2 | 0.01 | 7 | 10 | 29.1 | 0.02 |
| 3R | F | 4 | 0 |  |  |  | 3 | 5 | 43.7 | 0.02 |
| 4B | F | 1 | 0 | 2 | 0.0 | 0.00 | 5 | 11 | 30.2 | 0.02 |
| 4R | F | 4 | 1 | 1 | 0.0 | 1.00 | 1 | 1 | 0.0 | 0.01 |
| 5B | F | 1 | 3 | 2 | 29.2 | 0.01 | 3 | 7 | 22.9 | 0.02 |
| 5R | F | 4 | 5 | 7 | 70.5 | 0.02 | 2 | 8 | 29.5 | 0.02 |
| 6G | F | 1 | 1 | 3 | 61.5 | 0.01 | 18 | 17 | 106.7 | 0.02 |
| 6R | F | 4 | 3 | 2 | 0.0 | 0.01 | 2 | 8 | 92.9 | 0.02 |
| 7G | F | 1 | 4 | 6 | 133.0 | 0.01 | 6 | 13 | 47.4 | 0.02 |
| 7R | F | 2 | 2 | 3 | 22.3 | 0.01 | 5 | 8 | 51.4 | 0.02 |
| 8R | F | 2 | 3 | 4 | 24.0 | 0.01 | 12 | 11 | 71.3 | 0.02 |
| 0G | I | 2 | 11 | 9 | 25.8 | 0.01 | 8 | 6 | 11.4 | 0.02 |
| 1G | I | 2 | 11 | 12 | 22.0 | 0.02 | 4 | 5 | 38.5 | 0.02 |
| 2G | I | 2 | 6 | 9 | 25.3 | 0.02 | 5 | 7 | 21.9 | 0.02 |
| 3G | I | 2 | 5 | 8 | 38.5 | 0.02 | 3 | 5 | 1.5 | 0.02 |
| 4G | I | 1 | 1 | 4 | 31.2 | 0.01 | 8 | 12 | 48.2 | 0.02 |
| 5G | I | 1 | 6 | 9 | 10.3 | 0.02 | 7 | 8 | 84.1 | 0.02 |
| 8G | I | 1 | 10 | 10 | 193.5 | 0.02 | 5 | 9 | 20.0 | 0.03 |

**Table S5.** Number of affiliative and agonistic interactions performed between goats according to whether they are female or intersex.

|  | **Affiliative interactions**  Χ^2^_1_ = 38.4, P < 0.0001 | | **Agonistic interactions**  Χ^2^_1_ = 16.6, P < 0.0001 | |
| --- | --- | --- | --- | --- |
| **Receiver →**  **Emitter ↓** | **Female** | **Intersex** | **Female** | **Intersex** |
| **Female** | 39 | 8 | 100 | 22 |
| **Intersex** | 9 | 41 | 19 | 21 |

**Figure S1**. Picture of a 2 year-old intersex goat.

**Figure S2.** Picture of a 2 year-old female goat.
